# Exploring upper airway and laryngeal resistance to noninvasive ventilation in healthy awake adults

**DOI:** 10.14814/phy2.70621

**Published:** 2025-10-16

**Authors:** Anne Kristine Brekka, Maria Vollsæter, Zoe Fretheim‐Kelly, Manel Lujan, Roy Miodini Nilsen, Hege Havstad Clemm, John‐Helge Heimdal, Thomas Halvorsen, Ove Fondenes, Ola Drange Røksund, Tiina Maarit Andersen

**Affiliations:** ^1^ Thoracic Department Haukeland University Hospital Bergen Norway; ^2^ Faculty of Health and Social Sciences Western Norway University of Applied Sciences Bergen Norway; ^3^ Department of Paediatrics Haukeland University Hospital Bergen Norway; ^4^ Faculty of Medicine, Department of Clinical Medicine University of Bergen Bergen Norway; ^5^ Faculty of Veterinary Science Norwegian University of Life Sciences Oslo Norway; ^6^ Servei de Pneumologia Parc Taulí Hospital Universitari Sabadell Spain; ^7^ Institut d'Investigació i Innovació Parc Taulí (I3PT‐CERCA) Universitat Autònoma de Barcelona Sabadell Spain; ^8^ Department of Otolaryngology Haukeland University Hospital Bergen Norway; ^9^ Faculty of Medicine, Department of Surgical Science University of Bergen Bergen Norway

**Keywords:** induced laryngeal obstruction, noninvasive ventilation, translaryngeal resistance, upper airway resistance

## Abstract

The interaction between anatomical structures, pressure, and airflow impacts the airway resistance. The airflow during noninvasive ventilation (NIV) relies on the upper airway and laryngeal patency. This study aimed to quantify the airflow resistance at these levels during NIV. In this cross‐sectional study examining 10 healthy, awake adults, we established a NIV setup incorporating a continuous video‐recorded transnasal laryngoscopy and simultaneous airway pressure measurement using a transducer positioned above and below the vocal folds. Airflow and mask pressure were recorded by a pneumotachograph at the mask. NIV was delivered with inspiratory positive pressure (IPAP)/expiratory positive pressure (EPAP) set to 10/4 and 15/4 cmH_2_O. Upper airway (R_uaw_) and translaryngeal (R_tl_) resistance were calculated and compared with laryngoscopic findings. During IPAP10/EPAP4, the R_uaw_ was 4.25/4.21 and R_tl_ 2.20/3.45. During IPAP15/EPAP4, the R_uaw_ was 5.18/5.73, and the R_tl_ was 2.31/3.83. R_uaw_ was significantly higher than R_tl_ for inspiration at both IPAP levels (*p* = 0.001 and *p* = 0.012), and for expiration with IPAP15/EPAP4 (*p* = 0.048). The resistance appeared dynamic during the NIV cycle, and the findings aligned with the laryngoscopic observations. NIV modulates upper airway and translaryngeal resistance. Resistance increases with elevated IPAP levels, particularly within the upper airway.

## Graphical abstract

NIV modulates both upper airway and translaryngeal resistance. Our findings demonstrate that direct laryngoscopic observations align with the dynamic changes measured in resistance curves.

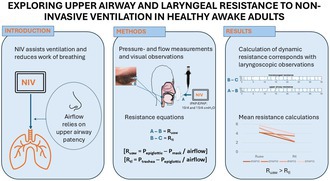

## 1 INTRODUCTION AND OBJECTIVES

Noninvasive ventilation (NIV) is an essential tool in respiratory support, delivering positive airway pressure to assist ventilation and reduce the work of breathing without the requirement for intubation or tracheostomy (Oppersma et al., 2013). Unlike invasive approaches, NIV depends on the functional integrity of the upper respiratory tract to maintain effective airflow. The upper respiratory tract refers to the anatomical region from the nasal cavity to the larynx and includes the upper airway (nasal cavity to below the epiglottis) and the translaryngeal segment (from the epiglottis to trachea) (Ferris Jr et al., 1964; Strohl et al., 2012). The upper respiratory tract accounts for a considerable portion of total airway resistance, even under normal physiological conditions in healthy individuals (Jounieaux, 1995a, 1995b). This resistance is shaped by complex interactions between rigid anatomical structures, soft tissues, dynamic muscle activity, and airflow. These flow–structure interactions influence airway patency and respiratory mechanics in ways that remain incompletely understood (Andersen et al., 2021; Ferris Jr et al., 1964; Strohl et al., 2012). A deeper understanding of these mechanisms is needed and clinically relevant. Patients in respiratory distress depend on various noninvasive devices to support breathing or coughing. These devices generally act by manipulating airflow and airway pressures—using positive pressure to assist inspiration and negative pressure to assist expiration—thereby interacting with the underlying flow–structure dynamics. If these interventions instead compromise airway patency, the devices will fail to function as intended.

During normal breathing, overall airway resistance ranges from 0.5 to 2.0 cmH_2_O/L/s (Briscoe & Dubois, 1958), with the larynx alone contributing 25%–50% of the resistance in the upper respiratory tract (Ferris Jr et al., 1964). These values have been established during normal breathing, and the introduction of positive pressure from NIV adds a new set of variables. NIV not only generates airflow through the airway but also imposes additional resistive loads due to the mask, the breathing circuit, and the patient's airway anatomy. It remains unclear how the upper respiratory tract adapts to this added load.

Previous studies have shown that applying varying levels of therapeutic pressure may elicit specific laryngeal responses, including dynamic narrowing consistent with what has been labeled inducible laryngeal obstruction (Andersen et al., 2013, 2017, 2018, 2022; Brekka et al., 2024; Georges et al., 2016; Jounieaux, 1995a, 1995b; Oppersma et al., 2013, 2019; Parreira et al., 1996; Sayas Catalán et al., 2017; Vollsæter et al., 2021). These findings suggest that positive pressure therapy can elicit structural and functional adaptations in the larynx. However, the exact implications of these adaptations for airflow resistance during NIV remain poorly understood.

Tailoring NIV to the individual patient requires an understanding of the anatomical and physiological interactions in the upper airway and larynx during positive pressure treatment (Conde et al., 2019; Oppersma et al., 2013; Sayas Catalán et al., 2017; Strohl et al., 2012). Obstructive responses, such as backward tongue displacement, epiglottic movements, or vocal fold adduction, can occur during both inspiratory and expiratory phases, and have been observed under various conditions in both healthy individuals and patients with neuromuscular conditions (Andersen et al., 2013; Andersen et al., 2017; Andersen et al., 2018; Jounieaux, 1995a; Jounieaux, 1995b; Sayas Catalán et al., 2017). Such responses can theoretically increase airflow resistance and potentially impair the effectiveness of the ventilatory support (Jounieaux, 1995a, 1995b). This highlights the importance of precise quantification of upper respiratory tract dynamics during NIV. Improved understanding of these mechanisms may enable more personalized adjustments of respiratory support devices, thereby enhancing comfort, optimizing ventilation, and ultimately improving compliance and long‐term adherence to therapy.

To bridge this gap, our study aimed to quantify airflow resistance at distinct anatomical levels during NIV in healthy individuals. Specifically, we sought to partition resistance between the mask and the epiglottis (upper airway) and across the larynx to the trachea. This study aims to elucidate the mechanisms underlying upper airway and laryngeal resistance during NIV, thereby providing insights to support optimized ventilatory strategies and tailored pressure settings in a clinical context.

## 2 PATIENTS AND METHODS

This was an explorative cross‐sectional observational study enrolling 10 healthy and awake participants recruited from the staff at Haukeland University Hospital, Bergen, Norway in the time period from June to September 2021. Exclusion criteria were age < 18 years, a history of laryngospasm or pneumothorax. The study was conducted in accordance with the amended Declaration of Helsinki (1964) and approved by The Regional Committee for Medical and Health Research Ethics in Western Norway (ID: 2021‐186684). Participants were well informed about the methods involved, and they had all signed informed consent forms.

Spirometry was performed (Vyntus spirometer, CareFusion, Hoechberg, Germany) according to the European Respiratory Society guidelines (Graham et al., 2019).

The NIV device VPAP Stellar (Resmed, San Diego, USA) was used to apply inspiratory (IPAP) and expiratory (EPAP) positive airway pressures, generating airflow. The participants were instructed to breathe normally for approximately 3 min with the NIV device in spontaneous mode. No additional humidity was provided. IPAP/EPAP was first set to 10/4 and after a five‐minute break to 15/4 cmH_2_O. Pressure was the only setting adjusted, whereas the remaining settings were kept unchanged (maximum time allowed for inspiration ranged from 0.5 – 3.0 s, medium trigger and cycle, risetime 200 ms, fall time 200 ms). The last 10 breaths were selected for analysis.

### 2.1 Data collection of the airflow and the three airway pressures to calculate resistance

**FIGURE 1:**
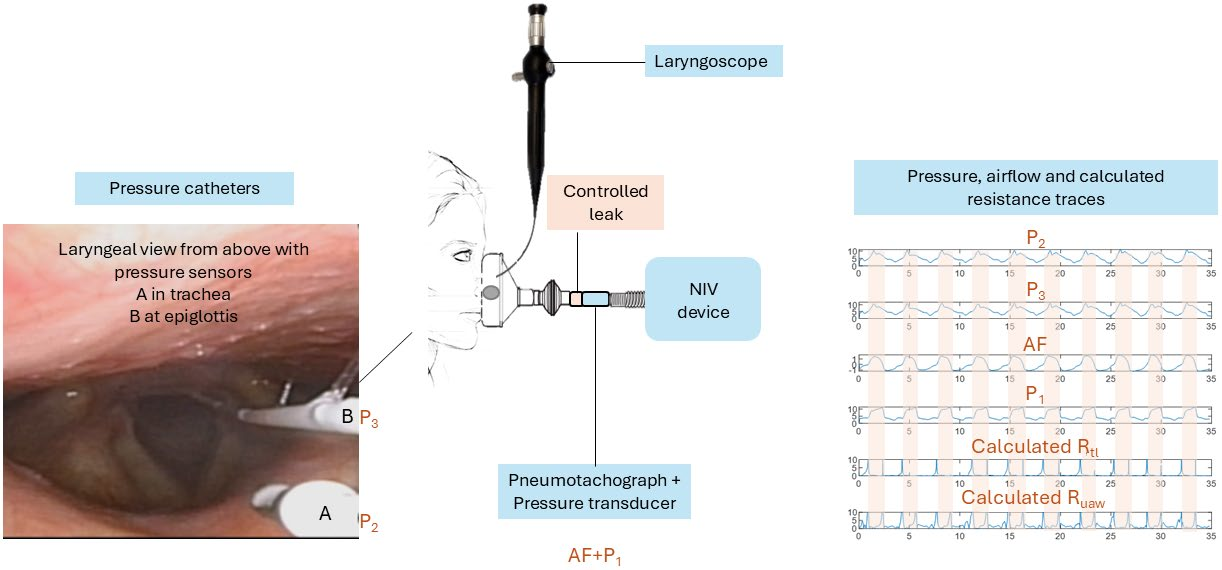
Setup of the examination with the recorded measurements and calculations. The NIV device delivers set pressures, the pneumotachograph (measuring airflow [AF] and pressure P_1_) is inserted in the circuit between the expiratory valve and the tube. Two additional pressure sensors are placed in the upper respiratory tract: (a) a pressure catheter ending at the tip of the epiglottis (measuring P_2_), and (b) a pressure catheter in the trachea (measuring P_3_). The calculated R_uaw_ and R_tl_ are shown, and the inspiratory phase is indicated in the traces. AF, airflow; NIV, noninvasive ventilation; P, pressure; R_uaw_, upper airway resistance; R_tl_, translaryngeal resistance.

To calculate resistance (R) in the upper airway and larynx, an experimental setup already used for previous measurements during other respiratory interventions (Andersen et al., 2024; Fretheim‐Kelly et al., 2019, 2022, 2023), including three pressures (P_1_, P_2_, and P_3_) and an airflow (AF) measurement, was used (Figure 1).

The upper airway and translaryngeal pressure were recorded with the participants sitting upright in a chair with their neck in a neutral position. A local anesthetic spray (0.1 mL of 4% Lidocaine, Accord Healthcare, Middlesex, United Kingdom) was applied before the lubricated laryngoscope (Olympus ENF‐VT3, Olympus, Japan) with a working channel providing a diameter of 4.9 mm was inserted through a modified full‐face mask (Facemask for Cough Assist Ventilatory Circuit, Respironics, Murrysville, USA) into the nasopharynx until a satisfactory view of the larynx was obtained (Andersen et al., 2013, 2017, 2018). The working channel was used to insert a spray‐tip catheter (Olympus, PW‐6C‐1, Olympus, Tokyo, Japan) to anesthetize the vocal folds and proximal trachea with a mist of Lidocaine (4%), as a preparation to place the tracheal and epiglottic pressure sensors. Additional doses were given as needed. The tracheal pressure catheter (Mikro‐Cath 825‐0101, Millar, Houston, USA) was introduced through the working channel of the laryngoscope and positioned approximately at the fifth tracheal ring (P_3_). Thereafter, the epiglottic pressure catheter was inserted and positioned at the tip of the epiglottis (P_2_) (Andersen et al., 2024; Fretheim‐Kelly et al., 2019, 2022, 2023), see Figure 1 for placements of pressure catheters. The examiner supported the laryngoscope manually. The pressure sensors were connected to a data‐acquisition box (Powerlab 8/35, AD Instruments, Oxford, United Kingdom), and the data were collected and stored on a laptop (Hewlett Packard, Palo Alto, California, USA) using LabChart 8.0 software (AD Instruments, Sydney, Australia). Data acquisition was set at 100 Hz. The laryngeal responses during the NIV intervention were video recorded.

Airflow (AF) and mask pressure (P_1_) were recorded during NIV using a pneumotachograph with a filter placed in the circuit (Citrex H5, IMT medical, Buchs, Switzerland), see Figure 1. Signals were sampled for analysis (FlowLab software, IMT medical, Buchs, Switzerland) and run on a computer with Microsoft Office Excel 2016 (Microsoft Corporation, Redmond, Washington, USA).

Retrospectively, all pressure‐, airflow‐, and calculated resistance traces were visually evaluated for evidence of interference using the software LabChart 8.0 (AD Instruments, Sydney, Australia) cycle by cycle. Data were validated by assessing the relationship between responses, pressure, airflow, and resistance curves, artifacts, drifts, and frequency responses. Upper airway resistance (R_uaw_) and translaryngeal resistance (R_tl_) were calculated using equations derived from the Hagen–Poiseuille law, based on measurements of airflow (AF) and three pressure readings (P_1_, P_2_, and P_3_), in accordance with the method described by Andersen et al. (2024).

$$R_{\mathrm{uaw}}=P_{2}–P_{1}/\mathrm{AF}$$

$$R_{\mathrm{tl}}=P_{3}–P_{2}/\mathrm{AF}$$

As the native flow includes both patient flow and intentional and unintentional leakage flows, the true patient flow was estimated by calculating the resistance at the transition point between the expiratory and inspiratory cycles using the following formula (Luján et al., 2019):

$$R=\text{EPAP}\;\left(\text{in}\;{\mathrm{cmH}}_{2}O\right)/\text{Flow}\;\left(\text{leakage}\right)$$

In the second step, the estimated patient flow was presented for the entire tracing as

$$\text{Patient flow}=\text{Native flow}–\left(\text{Pressure}\times R\right)$$

Finally, patient tidal volume was calculated by integrating the patient flow waveform. If the difference between the inspiratory and expiratory phases exceeded 10%, the cycle(s) were discarded from further analysis, as they were considered to involve unintentional asymmetric leaks.

The airflow values obtained during the procedures were presented as L/sec, the pressure as cmH_2_O, and resistance as cmH_2_O/L/sec.

The total amount (mL) of Lidocaine administered was recorded. The participants' perception of the examination was assessed by a numeric rating scale (NRS) of 0–10, where 0 indicated “not unpleasant at all” and 10 “the worst imaginable discomfort” after the examination.

The video clips from the laryngoscopy examination were reviewed and evaluated in real time and in slow motion as many times as needed by two experienced assessors to interpret the laryngeal responses (Andersen et al., 2013, 2017, 2018; Brekka et al., 2022).

### 2.2 Statistical analyses

Sample characteristics are reported as group medians with interquartile range. Descriptions of the laryngeal responses during NIV were reported as counts of participants.

To compare the R_uaw_ and R_tl_ during the IPAP and EPAP phases, we initiated our analysis by calculating paired differences (R_uaw_−R_tl_) for each of the 10 breaths for each of the 10 individual participants, resulting in a total of 100 paired differences. Subsequently, we computed the mean (and standard deviation) of these 100 paired differences and tested whether this mean was different from zero. This evaluation was conducted using linear mixed effects models, which accounted for the dependence of data within participants by allowing for unique intercepts for each participant. Given our small sample size, we used the restricted maximum likelihood estimation (REML) method and applied the Kenward‐Roger method to adjust for degrees of freedom (Rabe‐Hesketh & Skrondal, 2021). The same method was conducted to compare inspiration and expiration.

All statistical analyses were performed using Stata version 18 (StataCorp LLC, Texas, USA) for Windows. All *p* values were two‐sided, and values below 0.05 were considered statistically significant.

## 3 RESULTS

All 10 participants completed the study procedures and found them acceptable. Their sample characteristics, the amount of anesthesia, and their perception of the examination are given in Table 1. The setup resulted in a total of 200/200 NIV respiratory cycles without technical problems, which were used for analyses.

**TABLE 1:** Participant sample characteristics.

	Women *n* = 4	Men *n* = 6
Age, years	42 (32–45)	61 (26–62)
BMI, kg/m^2^	24.7 (23.3–25.6)	23.6 (23.1–26.9)
FVC, l	4.20 (3.71–4.64)	5.64 (4.76–5.99)
FVC, *z*‐score	1.5 (−0.4;1.7)	1.4 (0.5;1.1)
FEV1, L	3.16 (2.86–3.59)	4.6 (3.66–4.97)
FEV1, z‐score	0.6 (−0.7; 1.3)	0.6 (−0.4; 1.4)
PEF, L/sec	7.2 (6.8–7.7)	10.8 (8.6–14.1)
PEF, *z*‐score	−0.7 (−1.6;‐0.0)	−0.1 (−2.2;1.8)
Lidocaine, mL	5.6 (5.6–6.6)	5.5 (4.7–5.6)
Perception of (NRS)		
TFL (0–10)	3 (2–4.5)	3 (1–6)
TLP (0–10)	3 (2–5.5)	4.5 (2–6)
NIV (0–10)	2 (1.5–2.5)	2.5 (2–6)

*Note*: Data presented as median (interquartile range).

Abbreviations: BMI, body mass index; FEV1, forced expiratory volume in 1 s; FVC, forced vital capacity; NIV, noninvasive ventilation; NRS, numeric rating scale; PEF, peak expiratory flow; TFL, transnasal fiberoptic laryngoscopy; TLP, translaryngeal pressure.

The R_uaw_ was higher than the R_tl_ during inspiration and expiration, the differences were statistically significant during inspiration for IPAP10 and IPAP15, and for expiration during IPAP15/EPAP4. Both R_uaw_ and R_tl_ were higher with higher IPAP (Table 2).

**TABLE 2:** The calculated upper airway (R_uaw_) and translaryngeal resistance (R_tl_) in cmH_2_O/L/sec during inspiration and expiration.

NIV setting	R_uaw_	R_tl_	R_uaw_−R_tl_	*p* Value
IPAP + 10	4.25 ± 1.63	2.20 ± 1.55	2.04 ± 1.61	0.001
EPAP + 4	4.21 ± 1.73	3.45 ± 2.61	0.76 ± 2.55	0.359
IPAP + 15	5.18 ± 1.61	2.31 ± 3.05	2.87 ± 3.32	0.012
EPAP + 4	5.73 ± 2.44	3.83 ± 2.99	1.89 ± 2.89	0.048

*Note*: Data presented as mean ± SD. *p* Value for difference estimated by mixed effects model for clustered observations for difference between R_uaw_ and R_tl_.

Abbreviations: EPAP, expiratory positive airway pressure; IPAP, inspiratory positive airway pressure; NIV, noninvasive ventilation; R_uaw_, upper airway resistance; R_tl_, translaryngeal resistance.

**FIGURE 2:**
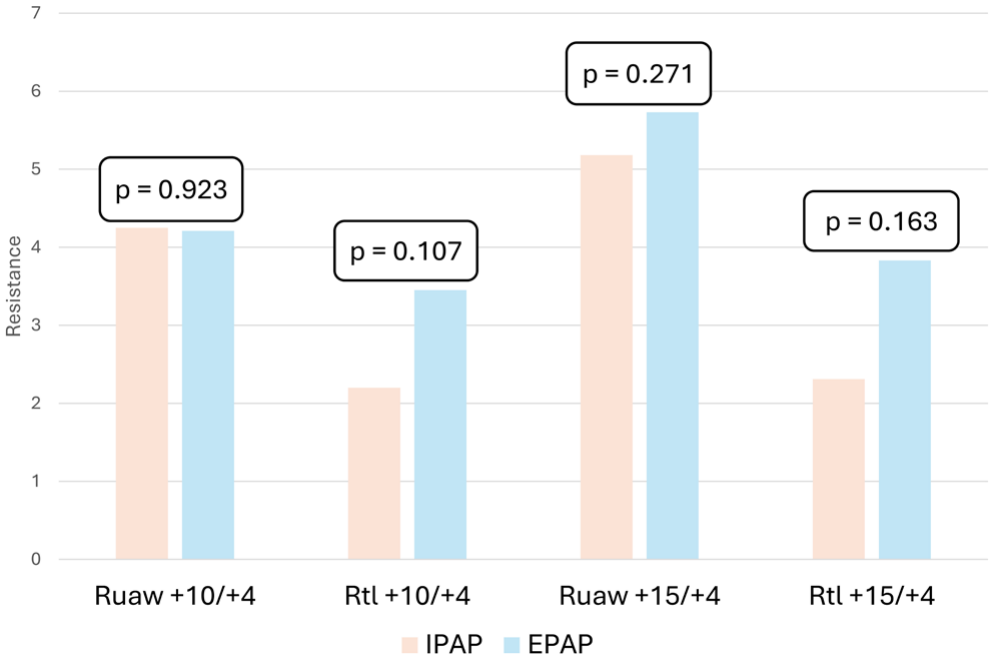
Comparisons of the upper airway (R_uaw_) and translaryngeal (R_tl_) resistance during inspiration versus expiration for the two NIV settings. The calculated mean ± SD in cmH_2_O is reported in Table 2, *n* = 10. EPAP, expiratory positive airway pressure; IPAP, inspiratory positive airway pressure; R_tl_, translaryngeal resistance; R_uaw_, upper airway resistance; *p* value for difference estimated by mixed effects model for clustered observations for differences between inspiration and expiration.

There was no statistical difference in resistance during inspiration versus expiration, although R_tl_ was consistently lower during IPAP (Figure 2).

### 3.1 Laryngoscopic findings

All participants had a structurally normal larynx at rest. During NIV treatment, all participants generally abducted both the vocal folds and the aryepiglottic folds during inspiration and adducted during expiration. In two participants, this represented the full range of observed movements. In the remaining eight participants, several additional movements were noted during inspiration that could potentially obstruct airflow. Three participants exhibited a tendency for adduction of the aryepiglottic folds, four demonstrated a high‐rising epiglottis, two showed a retroflex epiglottis for at least one breath with IPAP15, and two had a narrow hypopharynx. One participant swallowed during both interventions.

**FIGURE 3:**
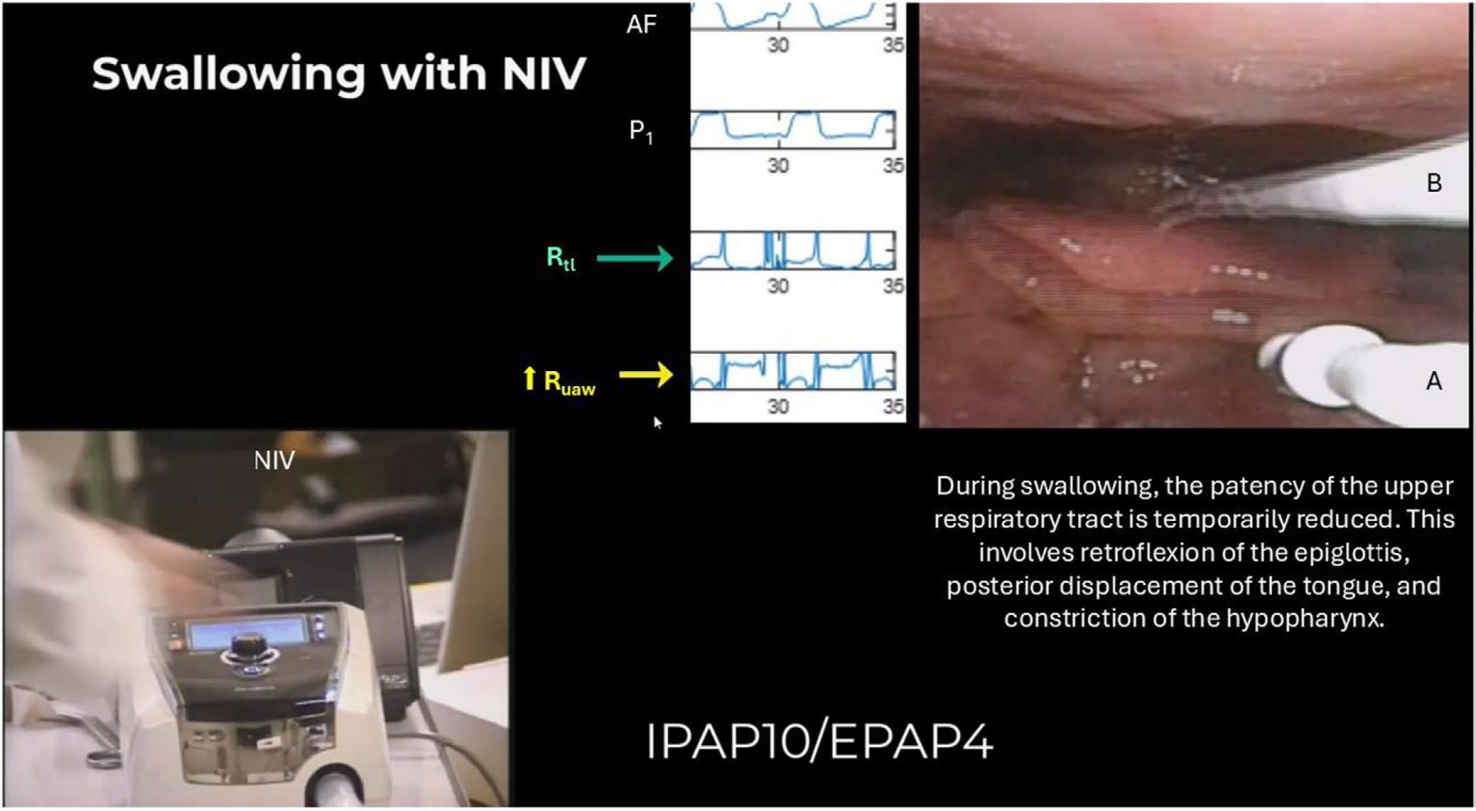
Still image from the Video S1. The figure shows one participant at the timepoint of swallowing, with airflow, device pressure (P_1_), R_tl_ (green arrow), and R_uaw_ (yellow arrow). R_uaw_ is elevated compared with participants without this observation. Pressure catheter placement is indicated: a, at the epiglottis, and b, in the trachea. Image/video published with participant consent. AF, airflow; EPAP, expiratory positive airway pressure; IPAP, inspiratory positive airway pressure; NIV, noninvasive ventilation; P, pressure; R_tl_, translaryngeal resistance; R_uaw_, upper airway resistance.

Qualitatively, both R_uaw_ and R_tl_ appeared dynamic during the respiratory cycle, with a peak at the initiation of both respiratory phases. As shown in Video S1/Figure 3, the observed laryngeal responses, as described above, affected R_uaw_ and R_tl_.

## 4 DISCUSSION

This is the first study to characterize resistance in the upper respiratory tract during noninvasive ventilation in healthy, awake adults, analyzing resistance separately across two key regions believed to play critical roles in modulating airflow capacity. Increasing IPAP led to higher resistances throughout the respiratory cycle, with R_uaw_ consistently exceeding R_tl_. The laryngoscopic observations aligned with the dynamic resistance curves, highlighting the role of the upper airway throughout the NIV cycle and demonstrating that the dynamics of laryngeal structures influence resistance.

Our findings indicate that resistance in the upper respiratory tract during NIV was higher than values reported during normal breathing (Baier et al., 1977; Ferris Jr et al., 1964; Savard et al., 1993). Increasing applied pressure increased resistance, a finding consistent with previous studies showing that R_uaw_ increases at higher pressures during mechanically assisted cough (Andersen et al., 2024) or NIV settings exceeding 20 cmH_2_O (Series et al., 1990). Interestingly, Series et al. (1990) showed that nasal resistance appears to contribute more to this increase than oropharyngeal resistance, further emphasizing the role of upper airway geometry in modulating pressure‐induced resistance. R_uaw_ was significantly higher than R_tl_ in our study, indicating that the upper airway segment contributes consistently to overall resistance during NIV. The increase in R_tl_ at IPAP15 suggests that higher IPAP also affects laryngeal structures, but to a lesser extent than the upper airway. This aligns with prior research showing that laryngeal resistance increases with higher airflow and inspiratory pressures (Andersen et al., 2024; Fretheim‐Kelly et al., 2022; Savard et al., 1993). However, this finding may appear counterintuitive, given that NIV is commonly used in clinical practice to reduce work of breathing and improve airway patency. The participants of this present study differ from the typical clinical population in which NIV is used, that is, patients with respiratory failure. However, reflexive airway narrowing has also been observed and described in patient populations (Conde et al., 2019; Oppersma et al., 2019; Sayas Catalán et al., 2017). While this does not necessarily contradict the beneficial effects of NIV in clinical populations, it highlights the complexity of upper airway behavior during positive pressure ventilation and underscores the need for individual titration of settings. These findings may be particularly relevant in patients who experience discomfort or intolerance to higher NIV pressures. A potential bias arises from measuring airflow at the mouth, as it assumes that all measured flow reaches the larynx. If actual laryngeal flow is lower due to compressible volume in the upper airway, R_tl_ is underestimated (Luján et al., 2019).

Airflow velocity is a key factor influencing airway resistance, with Reynolds number determining the transition from laminar to turbulent flow (Strohl et al., 2012). This present study used Hagen‐Poiseuille's equation, which is valid for laminar airflow, but the upper airway often exhibits turbulent airflow, which is better described by the Rohrer equation (Strohl et al., 2012). A R_uaw_ of 4.4 ± 0.6 cmH_2_O/L/s in healthy volunteers during NIV settings IPAP20/EPAP5 has been reported (Tuggey et al., 2007). This highlights a methodological limitation: Hagen‐Poiseuille's formula may underestimate resistance under turbulent conditions, as it does not account for the quadratic relationship between resistance and flow velocity in turbulent airflow.

Variation in resistance during the respiratory cycle is a normal physiological phenomenon. While few obstructive responses were observed among our healthy participants, even small adductive movements in the upper airway and larynx increased calculated resistance, emphasizing individual variability. Due to this large variation, we performed qualitative assessment of resistance curves, instead of formal statistical testing for difference.

The observed variations in airway resistance likely reflect a complex interplay of anatomical structures, physiological dynamics, and external influences, emphasizing the multifactorial nature of resistance under different conditions. This scenario is termed flow–structure interaction, a biomechanical phenomenon (Andersen et al., 2021; Tu et al., 2013). Airflow exerts forces on the flexible upper airway structures, causing deformations that alter airway geometry. These deformations, in turn, influence the airflow, creating feedback loops that shape the dynamic behavior of airway resistance. The upper respiratory tract is a dynamic cavity consisting of both rigid (nose and oral cavity) and compliant (pharynx and larynx) structures (Andersen et al., 2021), making the latter more prone to deformation under pressure (Oppersma et al., 2013). Flow–structure interaction models have been used in obstructive sleep apnoea (OSA) to show how positive pressure maintains upper airway patency and prevents collapse (Ashraf et al., 2022; Le et al., 2019). In NIV, similar principles apply; positive pressure prevents obstruction, supports gas exchange, and reduces respiratory effort (Oppersma et al., 2013). However, higher therapeutic pressures, as in IPAP15 versus IPAP10, increase the stress on flexible structures, causing deformation and airway narrowing, resulting in elevated resistance. The compliance of the pharynx and larynx makes these regions particularly susceptible to pressure‐induced resistance (Tu et al., 2013). Our findings suggest that while positive pressure is essential to maintain airway patency, it must be carefully titrated to balance the therapeutic benefits and minimize the biomechanical stresses. Excessive pressures can increase resistance and cause discomfort, potentially compromising NIV efficacy.

### 4.1 Strengths and limitations

This study demonstrated the feasibility of calculating airway resistance during NIV while at the same time integrating real‐time laryngoscopic visualization. The method coupled visual observations with physiological data. Using three measurement points enabled splitting airway resistance into two regions: R_uaw_—mask to epiglottis, and R_tl_—epiglottis to trachea. A similar setup has been performed during exercise (Fretheim‐Kelly et al., 2019, 2022, 2023), and at rest (Baier et al., 1977), and has also provided valuable information during mechanically assisted cough (Andersen et al., 2024).

Although the inclusion of only 10 healthy participants represents a limitation, this sample size is consistent with the approach in prior physiological studies (Jounieaux, 1995a, 1995b; Parreira et al., 1996; Savard et al., 1993; Series et al., 1990; Tuggey et al., 2007).

Including two IPAP levels provides insight into how resistance changes with pressure variations. However, the selected pressures, based on clinical practice for neuromuscular conditions, represent a limited range. Testing effects from higher pressures, commonly used in OSA and chronic obstructive pulmonary disease, or zero support could have made the findings applicable to an even broader range of individuals who benefit from NIV. Our findings, based on healthy individuals using a specific ventilator and the selected settings (Lalmolda et al., 2022), are limited by the inclusion of participants without clinical indications for NIV. This reduces the applicability of the results to patients with hypercapnia or hypoxia, which is associated with exaggerated laryngeal responses (Jounieaux, 1995a, 1995b). Moreover, laryngeal responses observed during wakefulness and in non‐supine positions may differ from those observed during sleep, which was not assessed in this study. Despite these limitations, the findings highlight variability in upper airway and translaryngeal resistance, reinforcing the importance of tailoring NIV settings to individual needs in clinical practice. This study serves as a strong incentive to pursue similar measurements in patient populations.

Although well‐rated by healthy motivated participants, the method is not yet ready for patient testing. The large volume of lidocaine used may affect both comfort and feasibility in clinical settings. Uncertainties remain regarding lidocaine's impact on the larynx and airway mechanics during NIV (Beydon et al., 1995). The observation that higher lidocaine doses reduced discomfort further supports the need for refinement.

Individual breaths were analyzed as independent measurements within each intervention. This was done to account for the dynamic nature of laryngeal responses, which can vary from breath to breath. By considering intraindividual variability in this way, we aimed to improve the repeatability and reliability of the measurements.

## 5 CONCLUSION

When using NIV, an interplay was observed between the applied flows and pressures and the anatomy of the upper airway and larynx, and these interactions were associated with variability in resistance across these segments. The upper airway and larynx played a role in modulating how the applied NIV pressures and airflows influenced airway resistance. Resistance was primarily affected by the level of IPAP, with the highest values observed in the upper airway and during the expiratory phase.

## AUTHOR CONTRIBUTIONS

AKB, MV, ZF‐K, HHC, J‐HH, TH, OF, ODR, and TMA conceived and designed research; AKB, MV, ZF‐K, HHC, J‐HH, TH, OF, ODR, and TMA performed experiments; AKB, ML, RMN, and TMA analyzed data; AKB, ML, RMN, and TMA interpreted results of experiments; AKB, ML, and TMA prepared figures; AKB, MV, ML, TH, OF, ODR, and TMA drafted manuscript; AKB, MV, ZF‐K, ML, RMN, HHC, J‐HH, TH, OF, ODR, and TMA edited and revised manuscript. All authors approved final version of the manuscript.

## FUNDING INFORMATION

This work was supported by the Western Norway Regional Health Authority, Bergen, Norway with grant number F‐12817‐D10980 (to AKB); and The Norwegian Advisory Unit for Home Mechanical Ventilation, Thoracic Department, Haukeland University Hospital, Bergen, Norway (to AKB); and The Western Norway Regional Health Authority General Research Fund for the HelpILO project (grant number F‐12654) with the equipment used under data collection. Financial support was their exclusive role.

## CONFLICT OF INTEREST STATEMENT

The authors have no perceived conflict of interest.

## Supporting information


Video S1.

## Data Availability

Due to privacy regulations (GDPR) and restrictions imposed by the Regional Committee for Medical and Health Research Ethics (REK), the datasets generated and analyzed during the current study are not publicly available. Data may be made available from the corresponding author on reasonable request, within the limits of ethical approval and participant consent.
